# *Garcinia dulcis* Fruit Extract Induced Cytotoxicity and Apoptosis in HepG2 Liver Cancer Cell Line

**DOI:** 10.1155/2015/916902

**Published:** 2015-10-18

**Authors:** Mohd Fadzelly Abu Bakar, Nor Ezani Ahmad, Monica Suleiman, Asmah Rahmat, Azizul Isha

**Affiliations:** ^1^Faculty of Science, Technology and Human Development, Universiti Tun Hussein Onn Malaysia (UTHM), Batu Pahat, 86400 Parit Raja, Johor, Malaysia; ^2^Institute for Tropical Biology and Conservation, Universiti Malaysia Sabah, Jalan UMS, 88400 Kota Kinabalu, Sabah, Malaysia; ^3^Department of Nutrition and Dietetics, Faculty of Medicine and Health Sciences, Universiti Putra Malaysia (UPM), 43400 Serdang, Selangor, Malaysia; ^4^Laboratory of Natural Products, Institute of Bioscience, Universiti Putra Malaysia (UPM), 43400 Serdang, Selangor, Malaysia

## Abstract

*Garcinia dulcis* or locally known in Malaysia as “mundu” belongs to the family of Clusiaceae. The study was conducted to investigate the anticancer potential of different parts of *G. dulcis* fruit extracts and their possible mechanism of action in HepG2 liver cancer cell line. MTT assay showed that the peel, flesh, and seed extracts of *G. dulcis* induced cytotoxicity in HepG2 cell line with IC_50_ values of 46.33 ± 4.51, 38.33 ± 3.51, and 7.5 ± 2.52 *µ*g/mL, respectively. The flesh extract of *G. dulcis* induced cell cycle arrest at sub-G_1_ (apoptosis) phase in a time-dependent manner. Staining with Annexin V-FITC and propidium iodide showed that 41.2% of the cell population underwent apoptosis after 72 hours of exposure of the HepG2 cell line to *G. dulcis* flesh extract. Caspase-3 has been shown to be activated which finally leads to the death of HepG2 cell (apoptosis). GC-MS analysis showed that the highest percentage of compound identified in the extract of *G. dulcis* flesh was hydroxymethylfurfural and 3-methyl-2,5-furandione, together with xanthones and flavonoids (based on literature), could synergistically contribute to the observed effects. This finding suggested that the flesh extract of *G. dulcis* has its own potential as cancer chemotherapeutic agent against liver cancer cell.

## 1. Introduction

In recent years, studies on the exploitation of natural compounds from fruits for medicinal purposes have received a great attention due to their health-promoting effects. Plants, particularly fruits and vegetables, contain a diverse source of phytochemicals such as phenolics. Daily consumption of fruits and vegetables has been shown to lower the risk of some chronic noncommunicable diseases such as cancer and cardiovascular diseases [1, 2]. Phenolic compounds have drawn increasing attention since they possess potent antioxidant potential and are abundantly present in fruits and vegetables and have been shown to exhibit anticancer effects. However, it is believed that synergistic or additive biological effects of multiple phytochemicals were involved rather than a single compound or a group of compounds that contribute to cancer treatment and prevention. It is estimated that more than 5000 phytochemicals have been identified and different antiproliferation mechanisms have been reported [3].

The latest world cancer report from International Agency for Research on Cancer (AIRC), a specialized cancer agency of the World Health Organization (WHO), has revealed that the liver cancer is now the second leading cause of death worldwide [4]. Liver cancer is caused by Hepatitis B virus (HBV) which passes from person to person through blood, semen, or other body fluids and can be marked by liver inflammation. The Sun Daily [5] reported that liver cancer in Malaysia (developing country) is on the rise and the fifth most common cancer affecting men where rates are more than twice as high in males as in females. Globally, it is also the seventh most commonly diagnosed cancer in adult women where up to a million people die each year from liver cancer. Therefore, early detection is desperately needed to prevent this cancer as this can be prevented by taking Hepatitis B vaccines: World's First Anticancer Vaccines. In 2006, only 27% of infants worldwide received the first dose of vaccines within 24 hours of birth. As of 2008, a total of 177 countries (91%) had introduced the HBV vaccine into their national infant immunization schedule as recommended by the WHO [6].

*Garcinia dulcis *belongs to the family of Clusiaceae (Guttiferae) and is a less common fruit, locally known as “mundu” among Sabahan or Ma-phut in Thailand. The fruit is endemic to Borneo, Java, the Philippines, and Thailand. It is a medium-sized tree, 5 to 20 m high, often with yellow latex in the fruits and white latex (turns to light brown on exposure to the air) in entire parts of the tree. The leaf is large and leathery with dark green colour. The fruit is spheroid, slightly pointed and turns to yellow-orange when ripe. There are usually 1 to 5 brown seeds embedded in the flesh. The skin of the fruit is thin and soft when ripe while the flesh is pulpy and yellow in colour. The flesh is slightly acidic but pleasant to eat [7, 8]. However, local people in Thailand used it to enhance the taste in cooking dishes or make pickles because the fruits are too sour to be eaten raw/fresh [9].

Previous literature reported that the leaf and seed of* G. dulcis* have been used traditionally for the treatment of lymphatitis, parotitis, and goitre whose effects may have been caused by endophytic fungi, known to be present in* Garcinia* spp. [10]. In Thailand, the stem bark has been used traditionally as anti-inflammatory agent while the fruit juice was used as expectorant. In addition to that, ripe fruit of* G. dulcis *contained at least 22 known compounds and two new compounds (Dulcisflavan and Dulcisxanthone B) [11]. These bioactive compounds played an important role as they might be a great contributor to treat various chronic diseases as reported in previous literature [12]. Therefore, the main objectives of this present study were to determine the anticancer potential of the 80% aqueous methanol extract of* G. dulcis* towards the proliferation of liver carcinoma (HepG2) cancer cell line* in vitro *and investigate their possible mechanism of action.

## 2. Materials and Methods

### 2.1. Plant Materials and Sample Preparation

The fruit of* G. dulcis *was collected from Tenom, Sabah, Malaysian Borneo, in November to December 2012. The plant sample was collected, identified, and deposited at BORNEENSIS Herbarium (BORH), Universiti Malaysia Sabah. The samples were then separated into peel, flesh, and seed. The small-cut pieces were stored at −80°C before being freeze-dried by using freeze dryer. The lyophilized samples were grounded to obtain fine powder. These ground samples were stored in air-tight plastic bag at −20°C for further analysis.

### 2.2. Sample Extraction

The extraction procedure was conducted according to Ikram et al. [13] with slight modifications. The samples were extracted using 80% methanol by mixing 0.05 g of lyophilized fruit powder with 15 mL solvent. The mixture was placed in a 100 mL beaker wrapped with aluminium foil and agitated at 200 rpm with the aid of orbital shaker for 2 hours. The mixture was then filtered through filter paper (Whatman number 4) in order to obtain a clear solution. The filtrate was then subjected to vacuum rotary evaporator at 40°C to remove methanol residue. The concentrated methanolic extract was freeze-dried to ensure the excess water was removed and crude extracts can be obtained.

### 2.3. Antiproliferative Assay

The MTT (3-(4,5-dimethylthiazol-2-yl)-2,5-diphenyltetrazolium bromide) assay was conducted based on the method described by Mosmann [14]. The HepG2 cancer cell line was first cultured in RPMI 1640 medium with L-glutamine and supplemented with 10% of fetal calf serum and 1% penicillin streptomycin in 75 cm^2^ flask and incubated at 37°C with 5% CO_2_ in a humidified atmosphere. The viability of cells was determined by staining with trypan blue. Exponentially growing cells were harvested and counted using haemocytometer. A specific culture medium was used to dilute the cells to a density of 1 × 10^6^ cells/mL. From this cell suspension, 100 *μ*L was pipetted into each well of a 96-well plate and incubated for 24 h in a 5% CO_2_ incubator at 37°C. The old media were discarded before the sample extracts were added with the highest concentration of 100 *μ*g/mL and make up the final volume of 100 *μ*L in each well. The plate was then incubated in 5% CO_2_ incubator at 37°C for 72 h. Then, 20 *μ*L of MTT reagent (3-(4,5-dimethylthiazol-2-yl)-2,5-diphenyltetrazolium bromide) was added to each well and left in incubator for 4 h. Subsequently, 100 *μ*L of solubilization solution (DMSO) was added to each well and reading was taken at wavelength of 570 nm using ELISA reader. The cytotoxicity percentage was determined using the following formula:

(1) $$\begin{array}{c} \%\text{cytotoxicity}=\frac{\text{optical density of sample}}{\text{optical density of control}}\times 100. \end{array}$$

The final results were expressed as inhibition concentration (IC_50_), the concentration of sample able to inhibit cell proliferation by 50% that was calculated graphically for each cell proliferation curve.

### 2.4. Cell Cycle Analysis

Cell cycle analysis was conducted according to Abu-Bakar et al. [15]. Cells at density of 1 × 10^6^ cells/mL were incubated with the extracts at IC_50_ value for 24, 48, and 72 h. All adhering and floating cells were harvested and transferred into sterile centrifuge tube and centrifuged at 1200 rpm for 10 min (4°C). The pellets were then washed with cold phosphate buffered saline (PBS) (10 mM sodium phosphate pH 7.2, 150 mM sodium chloride) and resuspended in 0.5 mL of cold PBS. Then, 5 mL of ice-cold 70% ethanol was added to the cell suspension and incubated at −20°C for 2 hours. Then, the cells were washed twice with cold PBS and centrifuged again at 1200 rpm for 10 min (4°C). After that, 500 *μ*L of propidium iodide/RNAse (10 *μ*g/mL propidium iodide containing 1 mg/mL RNAse) solution was added to the cells and incubated in the dark for 30 min at room temperature before being analysed by flow cytometer within 3 hours of staining.

### 2.5. Discrimination of Early and Late Apoptosis

The procedure was conducted according to methodology provided in Annexin V-FITC Apoptosis Detection Kit (catalog number APOAF, Sigma, USA). Cells at density of 1 × 10^6^ cells/mL were incubated with the extracts at IC_50_ value for 24, 48, and 72 h. All adhering and floating cells were harvested and washed twice with PBS before being transferred into sterile centrifuge tube. The cell pellet was then suspended in binding buffer (100 mM HEPES/NaOH, pH 7.5 containing 1.4 M NaCl and 25 mM CaCl_2_) at a concentration of ~1 × 10^6^ cells per mL. A sample (500 *μ*L) of this cell suspension was transferred to a 5 mL falcon tube, to which 5 *μ*L of Annexin V-FITC conjugate and 10 *μ*L of propidium iodide were added. The cells were incubated in the dark for 10 minutes at room temperature. The fluorescence of the cells was determined by flow cytometry.

### 2.6. Assay of Caspase Activity

Caspase-3 Colorimetric Assay (catalog number BF3100, R&D Systems) was used to determine the activity of caspase-3. Cells (~3 × 10^6^ cells) (treated and untreated) were collected and transferred into sterile centrifuge tube. The cells were centrifuged at 500 rpm for 5 min (25°C). Supernatant was discarded and 25 *μ*L of cold lysis buffer was added per 1 × 10^6^ cells. The cell lysates were incubated on ice for 10 min. Samples (50 *μ*L) of the lysate (supernatant) were aliquoted into 96-well microplate and 50 *μ*L of 2X reaction buffer 3 followed by 0.5 *μ*L of 10 mM DTT was added to sample wells. Then, 5 *μ*L of caspase-3 colorimetric substrate (DEVD-pNA) was added to each reaction well and incubated at 37°C for 2 h. Absorbance value was read at 405 nm using ELISA reader.

### 2.7. Gas Chromatography-Mass Spectroscopy (GC-MS)

The methanolic crude extract of flesh of* G. dulcis* was analysed by gas chromatography equipped with mass spectrometry (GC-MS-2010 Plus-Shimadzu). The column temperature was set to 50°C for 4 min, then increased to 320°C at the rate of 7°C/min, and then held for 20 min. The injector temperature was set at 280°C (split mode with the ratio being adjusted to 20 : 1, injection volume = 0.1 *μ*L). The flow rate of the helium carrier gas was set to 1 mL/min with total run time of 60 min. Mass spectra were obtained from the range* m/z* 40 to 700 and the electron ionization was obtained at 70 eV. The chromatograms of the sample were identified by comparing their mass spectra with the library data and the GC retention time against known standards (NIST 11 Library and Wiley Library).

### 2.8. Statistical Analysis

Data were analysed using SPSS software version 17.0 and reported as mean ± standard deviation. Analysis of variance (ANOVA) with Tukey test and independent sample *t*-test were conducted to identify the significant difference (*P* < 0.05). All the analysis was carried out in triplicate in three independent experiments.

## 3. Results and Discussion

### 3.1. Anticancer Potential of* G. dulcis* Extract on HepG2 Cancer Cell Line

MTT assay is a commonly used method to investigate the cytotoxicity of natural products. This assay is based on the ability of mitochondrial dehydrogenase enzymes from viable cells to cleave the tetrazolium rings of the pale yellow MTT and form dark blue formazan crystals which is largely impermeable to cell membranes, resulting in its accumulation within healthy cells. Thus, the absorbance of the solubilized formazan crystal is proportional to the number of living cells in the system [14].

The incubation of* G. dulcis *peel, flesh, and seed extract with HepG2 cancer cell line induced cytotoxicity with IC_50_ values of 46.33 ± 4.51, 38.33 ± 3.51, and 7.5 ± 2.52 *μ*g/mL, respectively (Table 1). Doxorubicin was used as positive control. The seed extract showed the lowest IC_50_ value, followed by flesh and peel. The lower the IC_50_ value, the more effective the anticancer potential of the pure compound or crude extract. Some edible fruit extracts have been previously reported to possess anticancer properties such as cranberry, lemon, apple, strawberry, red grape, banana, and grapefruit that showed potent antiproliferative activity towards HepG2 cell line [16], rowanberry, raspberry, lingonberry, cloudberry, arctic bramble, and strawberry towards HeLa (human cervical cancer) [17].

The inhibition of liver cancer cell lines can be partially explained by the presence of phenolic phytochemicals such as anthocyanins, phenolic acids, carotenoids, and flavonoids as well as xanthone compound that is mainly distributed in* Garcinia *species. According to Yang et al. [18], specific phytochemicals might act additively, synergistically, and/or antagonistically with other compounds to display antiproliferative activity. Previous study reported that high antioxidant level did not necessarily reflect the high anticancer effect of the plant extract and might be due to synergistic effect of the crude extract itself [19].

In addition to that, Mangostin (a type of xanthone compound) is also found in ripe fruit of* G. dulcis* where the exact compound can also be found in the pericarp of* G. mangostana* especially *α*-mangostin and *γ*-mangostin. Consumption of mangosteen pericarp extract (81%  *α*-mangostin and 16% of *γ*-mangostin) in ratio of 0.25% and 0.5% extract to food dosage in daily diet has been reported to inhibit tumor growth in HCT116 (human colorectal carcinoma) and reduce blood vessels in tumor towards Athymic NCr nu/nu mice [20]. Previous literature reported the occurrence of at least 24 compounds that have been identified in* G. dulcis* such as Dulcisflavan, Dulcisxanthone B, (-) epicatechin [11], and kaempferol (kaempferol 3,7-di-*O*-*α*-rhamnopyranoside) [21]. Epicatechin and kaempferol were two flavonoid compounds that belong to flavanols and flavonols, respectively. Kaempferol is known to possess antioxidant and anticancer activity which inhibit cell proliferation of MDA-MB-453 (human breast carcinoma) [22]. All these compounds might act synergistically to inhibit the proliferation of studied cancer cell lines.

### 3.2. Cell Cycle Distribution on the Flesh of* G. dulcis*

**Figure 1:**
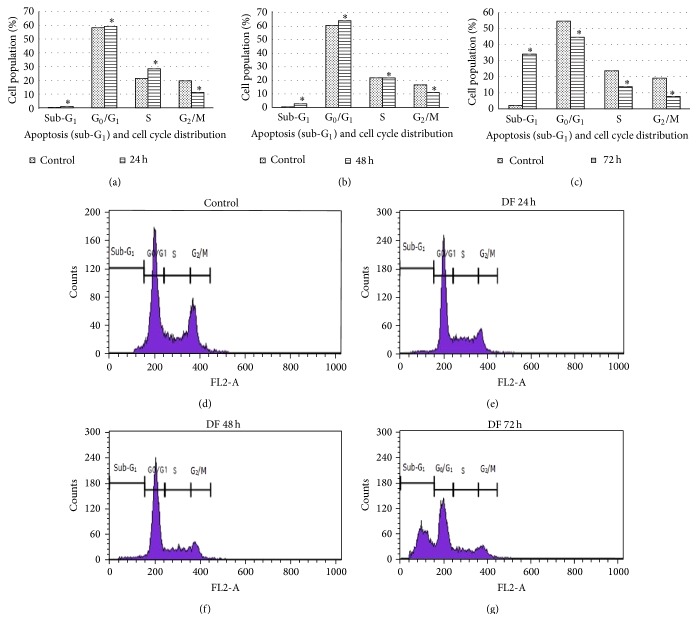
Cell cycle analysis of HepG2 cancer cell treated with the methanolic flesh extract of* G. dulcis *at IC_50_ value. The distribution of cells undergoing apoptosis in various phases of the cell cycle was determined at 24 hours (a), 48 hours (b), and 72 hours (c) in comparison to the respective control. The values are presented as mean ± standard deviation, where *∗* indicated significant difference relative to control (*P* < 0.05). The representative flow cytometric scans of untreated HepG2 cells and those treated with methanolic flesh extract of* G. dulcis* for 24, 48, and 72 hours are presented in Figures (d) to (g), respectively.

Due to the fact that only the flesh part of the fruit can be consumed, and also the seed extract induced cytotoxicity in normal mouse fibroblast cell line (data not shown), further experiment on cell cycle distribution and apoptosis-inducing effect of* G. dulcis *only proceeded with the flesh crude extract of* G. dulcis *using the IC_50_ value of 38 *μ*g/mL. Apoptosis and cell cycle arrest in HepG2 cancer cell were studied after exposure to* G. dulcis* flesh extract at IC_50_ concentration for 24, 48, and 72 hours (Figure 1). Flow cytometric analysis (using RNAse and propidium iodide) was conducted to measure the DNA content of HepG2 cancer cell which allows us to identify whether the growth inhibitory effect of the fruit extract was caused by specific perturbation of cell cycle-related events.

The cell cycle analysis demonstrated that the flesh of* G. dulcis* caused an accumulation of cells in the G_0_/G_1_ phase. This causes the inhibition transition of cells towards S phase and allowed the increase in the proportion of cells in sub-G_1_ phase. An obvious accumulation of cells in sub-G_1_ phase can be observed from 2% in control to 34% after 72 hours. The increase in sub-G_1_ phase was a reflection of apoptosis induction. Gradual decrease in G_0_/G_1_ phase, S phase, and G_2_/M phase can be observed from 24 h to 72 hours as compared to control. This condition might be due to the inhibition of DNA replication affected by the inability of the cells to replicate damaged DNA caused by the sample extracts, condensation of chromatin, and nuclear fragmentation [23]. The treatment of SK-MEL-28 (human melanoma) cells with *α*-mangostin significantly increased the sub-G_1_ peak with a concomitant decrease in G_1_ phase, indicating induction of apoptosis [24].

### 3.3. Early and Late Apoptosis

Apoptosis is a physiological process of killing cells and is an important process to eliminate tumours. The apoptosis process can be characterized by membrane blebbing (without loss of cell integrity), shrinkage of cells and nuclear volume, chromatin condensation, DNA fragmentation, and formation of membrane-bound vesicles (apoptotic bodies) which can be triggered by multiple independent pathways, from within or outside the cell [25].

The Annexin V-FITC Apoptosis Detection kit is the combination of fluorescein isothiocyanate (FITC) and annexin V with propidium iodide to distinguish living cells in early and late apoptosis. The annexin is a group of homologous proteins which bind phospholipids in the presence of calcium. During early apoptosis, phosphatidylserine which is usually located in the inner membrane of cells is transported into the outer portion of the membrane which can be detected by its strong affinity for Annexin V-FITC whereas the dead cells can be detected by the binding of propidium iodide to the cellular DNA in cell [15].

**Figure 2:**
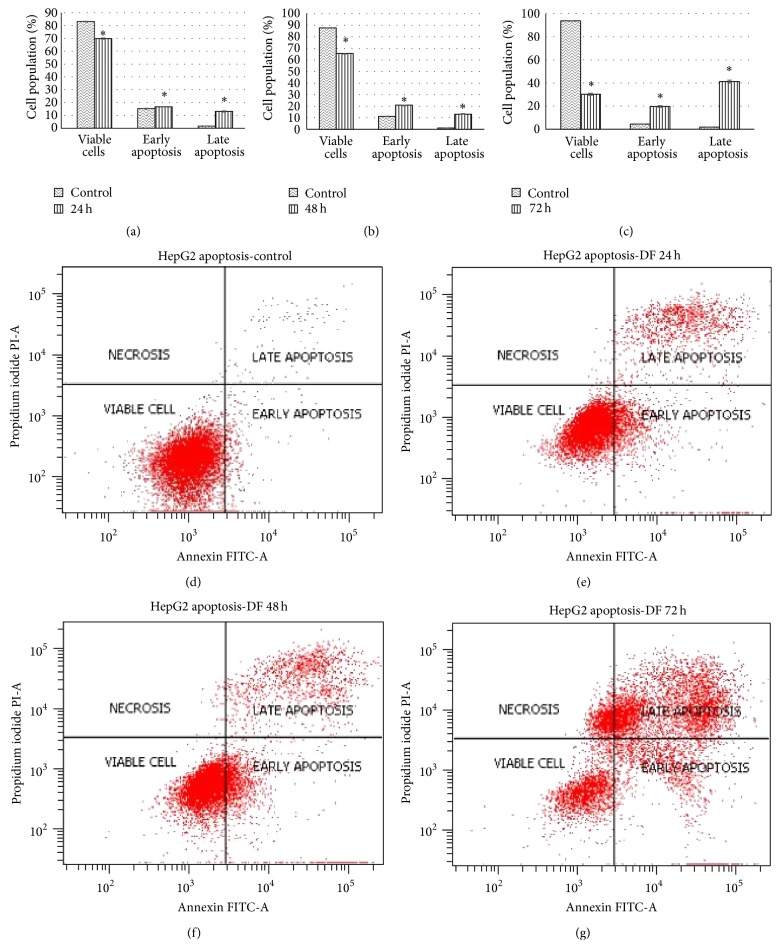
Apoptosis study upon treatment of the methanolic flesh extract of* G. dulcis* at IC_50_ value. The distribution of cells undergoing early and late apoptosis together with viable cells was determined at 24 hours (a), 48 hours (b), and 72 hours (c) in comparison to respective control, using Annexin V-FITC and propidium iodide flow cytometric analysis. The values are presented as mean ± standard deviation, where *∗* indicated significant difference relative to control (*P* < 0.05). The representative flow cytometric scans of untreated HepG2 cells and those treated with methanolic flesh extract of* G. dulcis* for 24, 48, and 72 hours are presented in Figures (d) to (g), respectively.

**Figure 3:**
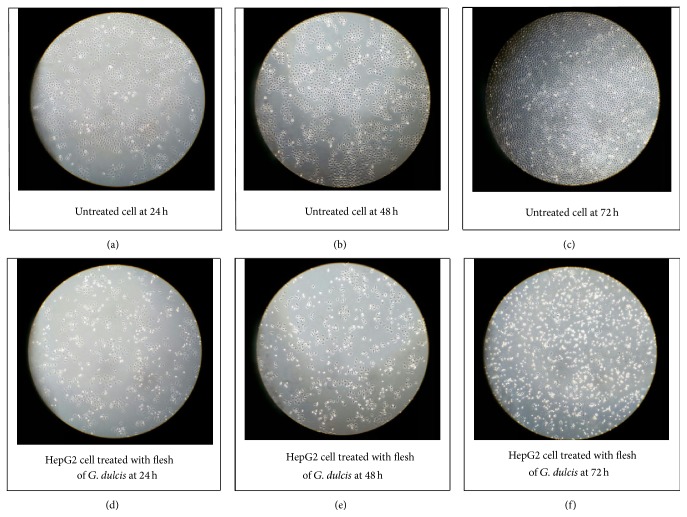
Morphological changes of HepG2 cancer cell line upon treatment with the flesh of* G. dulcis* at (d) 24 h, (e) 48 h, and (f) 72 h compared to untreated cell (a), (b), and (c).

The result showed that there was significant reduction in the proportion of cells in the viable cell group when treated with methanolic flesh extract of* G. dulcis *(Figure 2). These were concomitant with an increase in the proportion of cells in early and late apoptosis for 24, 48, and 72 hours of treatment when compared to control. The total values of apoptosis increased up to 29.8%, 34.0%, and 60.9%, respectively. At 24 hours, the values of viable cell started to decline by 69.8% as compared to control, with significant increase in early and late apoptosis with the values of 16.7% and 13.1%. The same trend of reduction in proportion of viable cells and increase of early and late apoptosis were observed for 48 hours. At the end of 72 hours, the total apoptosis increased up to 60.9% which comprise of early apoptosis (19.7%) and late apoptosis (41.2%). Some of the cells induce cell death through necrosis. Necrosis is always considered to be almost “accidental death” that may cause death of the cell at random and uncontrollably. The morphological changes of the cell undergoing apoptosis were shown in Figure 3. The induction of apoptosis was confirmed through activation of caspase.

### 3.4. Caspase Activity

**Figure 4:**
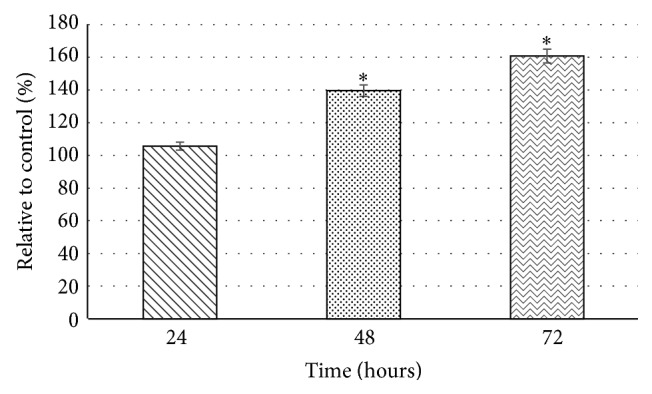
Activation of caspase in HepG2 cancer cell after incubation with methanolic flesh extract of* G. dulcis* at IC_50_ value for 24, 48, and 72 hours. The values are presented as mean ± standard deviation, where *∗* indicated significant difference relative to control (*P* < 0.05).

Caspase-3 is the most prevalent caspase within cells and responsible for most of apoptotic effects and upon activation, it was able to induce Poly ADP Ribose Polymerase (PARP) cleavage and DNA break and finally leads to apoptosis. The exposure to the flesh of* G. dulcis* towards HepG2 cancer cell line led to the activation of caspase-3 (Figure 4). This activation confirms that one of the initiator caspases (caspase-2, caspase-8, and caspase-9) was first activated which eventually could activate the executioner caspase (caspase-3, caspase-6, and caspase-7) which in this case was caspase-3 and thus led to the induction of apoptosis [26]. Caspase-3 was responsible for DNA fragmentation and morphological changes associated with apoptosis whereas caspase-2 and caspase-9 were early biomarkers of apoptosis which act as downstream targets of cytochrome c release from mitochondria which will then activate caspase-3 [27]. This finding was in agreement with previous study [28] where *α*-mangostin was able to suppress cell viability and colony formation which caused cell cycle arrest upon activation of caspase-3 towards PC3 and 22Rv1 (human prostate) cancer cell line. Induction of apoptosis can be observed in COLO205 (human colorectal adenocarcinoma) when exposed to 48 mg of *α*-mangostin and 6.40 mg of *γ*-mangostin per gram extract where the apoptosis induction happened at caspase-3 and caspase-8 pathway [29].

### 3.5. Gas Chromatography-Mass Spectroscopy (GC-MS)

**Figure 5:**
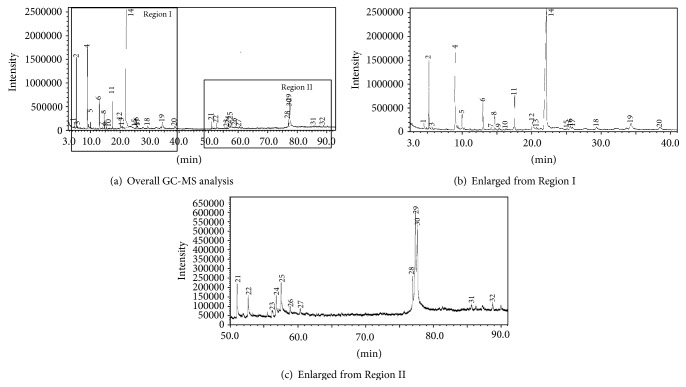
Chemical compounds of methanolic crude extract of* G. dulcis *detected using GC-MS, with relative retention time (^*t*^ *R*); (a), (b) enlarged from Region I and (c) enlarged from Region II.

Methanolic crude extract of flesh of* G. dulcis *was subjected to GC-MS analysis. GC-MS analysis is a widely used method that combines the features of gas chromatography and mass spectrometry purposely to identify compounds in a sample (Figure 5). The identification and characterization of chemical compounds of* G. dulcis* extracted in 80% aqueous methanol are shown in Table 2. The highest percentage of compound identified in the crude extract of* G. dulcis *was hydroxymethylfurfural or 5-hydroxymethylfurfural (HMF).

HMF is an organic compound, absent naturally in food but generated in sugar-containing food during heat-treatments like drying or cooking. This compound was commonly found in bakery products, malt, fruit juices, and coffee, with extremely high concentration in some food items such as dried fruits, caramel, and vinegar [30]. HMF was used as marker of quality in processed fruits, coffee, honey, and milk as well as monitoring the heating processes applied to cereal products such as pasta drying, bread baking, bread slices toasting, and extrusion of baby cereals and breakfast cereals [31]. HMF might occur naturally in the fruit or be generated during high temperature processes such as drying or during GC-MS analysis.

Previous study reported that the HMF compound showed cytoprotective effect by protecting LO2 (normal liver cell line) cell against cytotoxicity induced by hydrogen peroxide (H_2_O_2_). Hydrogen peroxide is a major precursor of highly reactive free radicals and it has been reported to decrease the viability of cell and induce apoptosis in many cells such as cardiomyocytes, chondrocytes, tendon fibroblasts, hepatocytes, and human umbilical vein endothelial cell. The compound significantly inhibits the effect of cell apoptosis in a dose-dependent manner after incubation of the cell with 1 *μ*g/mL HMF for 24 hours and thus decreased caspase-3 activity as well as nitric oxide level [32]. HMF which is also present in roasted coffee residue suppressed cytotoxicity in mouse embryonic fibroblast cell which is induced by hydrogen peroxide [33]. Liu et al. [34] also reported that HMF increases enzyme activities such as superoxide dismutase (SOD) and glutathione peroxidase (GPx) as well as potential therapeutic agent for the treatment of Alzheimer's disease.

In comparison to previous study by Haroun et al. [35], three out of 10 compounds identified in the pomegranate peels extract were also found in methanolic crude extract of flesh of* G. dulcis, *namely, HMF, n-hexadecanoic acid, and octadecanoic acid. These compounds were believed to contribute to antimicrobial effects. Previous research revealed that most of predominate compounds identified in crude extract through GC-MS analysis are biologically active molecules and considered to be part of plant defence system [36]. Hence, it is possible that these compounds present in methanolic crude extract of flesh of* G. dulcis* contributed to the antioxidant and anticancer properties.

## 4. Conclusion

In conclusion, the methanolic flesh extract of* G. dulcis* has demonstrated promising anticancer properties against liver cancer. Increasing awareness, promotion, and utilization of this fruit for public benefits are highly encouraged. Proteomic, genomic,* in vivo*, and human clinical studies are needed to determine the efficacy of the fruit extract to serve as natural cytotoxic agent in cancer patient.

## Figures and Tables

**Table 1:** IC_50_ value of samples tested against HepG2 cancer line by using MTT.

Parts	IC_50_ value (mean ± SD)
Peel	46.33 ± 4.51
Flesh	38.33 ± 3.51
Seed	7.5 ± 2.52
Doxorubicin	2.0 ± 0.87

SD: standard deviation; *n* = 3.

**Table 2:** Secondary metabolites in the *G. dulcis* flesh extract based on GC-MS analysis.

Peak	RT	Compound name	%
14	22.175	5-Hydroxymethylfurfural	39.61
4	9.062	2,5-Furandione, 3-methyl-	26.24
2	5.263	Furfural	6.65
13	20.732	1-Butanol, 2-methyl-, propanoate (CAS) 2-methylbutyl propionate	0.54
12	20.130	Catechol	4.35
6	13.024	2,5-Furandione, dihydro-3-methylene-	3.53
11	17.579	4H-Pyran-4-one, 2,3-dihydro-3,5-dihydroxy-6-methyl-	3.33
8	14.697	Furyl hydroxymethyl ketone	3.09
19	34.318	D-Allose	2.68
5	10.047	2,4-Dihydroxy-2,5-dimethyl-3(2H)-furan-3-one	1.20
20	38.486	1,6-Anhydro-.alpha.-d-galactofuranose	1.16
22	52.692	5,5′-Oxy-dimethylene-bis(2-furaldehyde)	0.94
1	4.556	1,4-Dioxadiene	0.59
7	14.198	1,3,5-Triazine-2,4,6-triamine	0.49
21	51.056	n-Hexadecanoic acid	0.36
9	15.298	1,3,5-Triazine-2,4,6-triamine	0.33
25	57.555	Octadecanoic acid	0.32
24	56.821	Heptadecene-(8)-carbonic acid-(1)	0.27
3	5.713	2-Furanmethanol	0.14

RT: retention time.
